# Perceived Effectiveness and Motivations for the Use of Web-Based Mental Health Programs: Qualitative Study

**DOI:** 10.2196/16961

**Published:** 2020-07-31

**Authors:** Heidi Eccles, Molly Nannarone, Bonnie Lashewicz, Mark Attridge, Alain Marchand, Alice Aiken, Kendall Ho, Jianli Wang

**Affiliations:** 1 Work & Mental Health Research Unit The Institute of Mental Health Research University of Ottawa Ottawa, ON Canada; 2 Department of Community Health Sciences, Cumming School of Medicine University of Calgary Calgary, AB Canada; 3 Attridge Consulting, Inc Minneapolis, MN United States; 4 School of Industrial Relations University of Montreal Montreal, QC Canada; 5 Public Health Research Institute University of Montreal Montreal, QC Canada; 6 Office of Research and Innovation Dalhousie University Halifax, NS Canada; 7 Department of Emergency Medicine University of British Columbia Vancouver, BC Canada; 8 Shandong Key Laboratory of Behavioral Medicine School of Mental Health Jining Medical University Jining China; 9 School of Epidemiology and Public Health University of Ottawa Ottawa, ON Canada; 10 Department of Psychiatry University of Ottawa Ottawa, ON Canada

**Keywords:** prevention, mental health, depression, cognitive behavioral therapy, motivators, perceived effectiveness, internet-based intervention, interview

## Abstract

**Background:**

The prevalence of depression is high and has been stable despite increased treatment, research, and dissemination. People encounter barriers to seeking traditional mental health services, which could be mitigated by using web-based prevention methods.

**Objective:**

This study aims to understand what people at high risk for depression perceive as effective aspects of web-based mental health programs and what motivates people at high risk for depression to use web-based mental health programs.

**Methods:**

We conducted an inductive content analysis using telephone interview data from 77 participants at high risk for depression who were recruited from 2 randomized controlled trials (RCTs). Participants from the first RCT were working men who had been randomly assigned to 1 of the following 3 groups: control group, who had access to general depression information from a website called BroMatters; intervention group 1, who had access to the BroMatters website along with the associated BroHealth web-based mental health program; and intervention group 2, who had access to the BroMatters website, the BroHealth web-based mental health program, and telephone sessions with a life coach. Participants from the second RCT were men and women who had been assigned to the intervention group, who received access to the HardHat web-based mental health program, or the control group, who only received access to the HardHat web-based mental health program following completion of the RCT. Participants for this inductive content analysis study were recruited from the intervention groups in both RCTs. Two groups of participants (n=41 and n=20) were recruited from the BroHealth RCT, and a third group comprised 16 participants that were recruited from the HardHat RCT.

**Results:**

We generated four categories regarding the perceived effectiveness of web-based programs and five categories related to what motivates the use of web-based programs. Participants identified awareness, program medium and functionality, program content, and coaches as categories related to the effectiveness of the programs. Categories of motivators to use web-based programs included providing reminders or incentives, promotion of the programs, providing appropriate medium and functionality, appropriate content, and perceived need. The final category related to motivators reflects perceptions of participants who were either unsure about what motivates them or believed that there is no way to motivate use.

**Conclusions:**

Conflicting evidence was obtained regarding the perceived effectiveness of aspects of the content and functionality of web-based programs. In general, web-based mental health programs were perceived to help increase mental health awareness, especially when it includes live access to a coach. However, the results also revealed that it is difficult to motivate people to begin using web-based mental health programs. Strategies that may motivate the use of such programs include perceived personal need, effective promotion, providing incentives and reminders, and improving functionality.

## Introduction

### Background

Depression is a prevalent mental health disorder [1], is associated with an increased risk of suicide [2], and has comorbidities with multiple physical health conditions such as cardiovascular diseases [3], diabetes [4], and fibromyalgia [5]. Depression also impacts the economy, costing employers US $44 billion every year in the United States [6]. Globally, workplace stress can cost between US $220 million and US $200 billion per country [7]. Despite the growth in treatments for depression [8-10], the rate of depression has remained stable for more than 10 years [11,12]. Thus, treatment alone is unlikely to substantially reduce the prevalence and incidence of depression [13]; instead, a focus on prevention is necessary. Indeed, in a recent Alberta-based research project examining views from a range of mental health stakeholders, preventative measures were identified as a top priority for future depression research [14,15]. Preventing the occurrence or delaying the onset of depression can decrease symptoms [16] and reduce the burden for employers [17].

Barriers to seeking help related to treating and preventing depression are numerous and include stigma and availability of and access to services [18,19]. Web-based methods for treating and preventing depression have the potential to mitigate these barriers; in fact, web-based programs have been shown to be as effective as traditional methods [20-22] and web-based education tools [23]. Facilitators reported in the literature about web-based mental health programs include ease of website navigation and user characteristics such as curiosity about web-based mental health programs [24]. Web-based methods may be especially beneficial in rural communities where self-sufficiency is highly valued and access to therapeutic treatment may be particularly limited [25]. However, the usage of web-based mental health programs remains low. A study comparing face-to-face and web-based program usage reported completion rates of 84% and 65%, respectively [26]. Other studies of web-based mental health programs offered participants access to the program beyond the study time frame and reported that approximately 51% of participants wished to have continued access. Of those given continued access, only 38% went on to log on to the program [27].

People are often concerned about privacy and confidentiality for fear of being stigmatized for accessing mental health services [28]. These concerns may leave people reluctant to use web-based mental health programs. Furthermore, given the relative novelty of web-based mental health programs, many people are skeptical of program effectiveness for reducing or preventing depression compared with face-to-face therapies and, correspondingly, are less likely to use such programs [28,29]. However, few studies have investigated how users perceive web-based mental health programs for depression prevention [30].

### Objectives

In view of user skepticism and the need for a fuller understanding of user perceptions of web-based mental health programs, our purpose is to identify factors that motivate use (initiation and adherence) of such programs and the perceived effectiveness of the programs. We achieve our purpose by analyzing data drawn from qualitative interviews conducted with a sample of web-based mental health program users who are at high risk of developing depression.

## Methods

### Research Design

This qualitative study complements 2 randomized controlled trials (RCTs). The RCTs were aimed at understanding strategies for preventing depression and evaluating user perceptions of the effectiveness of web-based mental health programs. This qualitative component elaborates on the understanding derived through the RCT studies by illuminating what users perceive as effective about web-based mental health programs and what motivates the use of such programs. In using a qualitative approach, we align ourselves with researchers who highlight the power of qualitative research to support contextual understanding of complex mental health phenomena [31] and to engage users in determining how to implement findings with respect to mental health in real-life settings [32].

We studied the perceived effectiveness of web-based mental health programming and what motivates the use of web-based mental health programs, and we treated these topics as emerging and yet to be coherently understood. Accordingly, we employed an inductive content analysis approach [33] of data collected through telephone interviews. Although we had some predetermined goals, our study was inductive in 3 important ways: (1) although we had always planned to study user perceptions of web-based mental health program effectiveness, our decision to study what motivates the use of web-based mental health programs emerged as we noted trends of nonuse among our RCT sample; (2) correspondingly, we selected participants because they were nonusers; and (3) the findings we present were derived from the data and not in accordance with predetermined categories.

Below, we outline the broader RCT studies from which participants for this inductive content analysis study were drawn. We follow this with a description of our qualitative sample, data collection, and data analysis, including trustworthiness measures.

### Broader RCT Study Context: BroHealth and HardHat

#### BroHealth RCT

The BroHealth RCT was about participants’ perceptions of the effectiveness of the BroHealth web-based program, which was designed to prevent depression in working men who are at high risk of having a major depressive episode (MDE) in the next 4 years. BroHealth includes modules that provide information about stress and depression, web-based self-checks that help monitor symptoms, and a variety of self-help components such as cognitive behavioral therapy (CBT) and goal setting and tracking. Qualified coaches were also available to participants for 40-min sessions to a maximum of once per week. Coaches help participants set goals and help them work through problems. The study protocol from which BroHealth was developed is available elsewhere [34].

BroHealth RCT participants were recruited across Canada using the random digit dialing method. Participants were men who were (1) older than 18 years, (2) working for pay, and (3) at high risk for an MDE determined by the depression risk calculator developed and validated by Wang et al [35] but who had not had an MDE in at least two months. Participants were also required to be able to communicate in English or French and have access to a telephone and the internet. BroHealth RCT participants were interviewed at baseline, at 6 months, and at 12 months, and it was after the baseline interview that participants were randomized into the control group or into 1 of the 2 intervention groups:

1. Control group: participants received general information about depression through the BroMatters website
2. Intervention group 1: participants received general information about depression through the BroMatters website as well as access to the associated BroHealth web-based program
3. Intervention group 2: participants received general information about depression through the BroMatters website, access to the BroHealth program, and access to a qualified coach with whom they could schedule telephone sessions.

Every other week, participants received reminders via email to encourage their engagement with the BroHealth program. Participants received incentives (about Can $25 [US $19] gift cards) for completing each interview, but they did not receive any incentive for completing the web-based program.

#### HardHat RCT

The HardHat RCT was about participants’ perceptions of the effectiveness of the HardHat web-based program designed to prevent depression in both men and women who are at high risk of having an MDE in the next 4 years. The HardHat program is coach assisted and comprises 5 compulsory and 4 supplementary sessions, which include short homework and in-class assignments. The 5 compulsory sessions had to be completed sequentially, and finishing one would unlock the next session. The 4 supplementary sessions could be accessed at any point in time. The sessions entail problem-solving therapy and components of CBT. Coaches had a minimum of a Bachelor of Science degree and had attended an intensive 2-day training session provided by a psychiatrist with CBT expertise. Coaches answered questions, guided users through the program, and provided feedback on homework and assignments. Coaches met weekly to discuss the participants’ cases and any issues encountered during coaching. Coaches also met with the psychiatrist on a quarterly basis.

HardHat RCT participants were recruited through a partnership with Green Shield Canada, a not-for-profit Canadian health and dental insurance company. An advertisement and link were posted on the Green Shield Canada health portal. Prospective participants could click the link and complete the eligibility questionnaire, which included a depression risk assessment using the same risk calculator used with participants in the BroHealth RCT [34]. Participants were men and women who were aged 18 years or older and at high risk of having an MDE in the next 4 years or had experienced an MDE in the past but who had not experienced symptoms for at least two months. Participants also needed to be able to communicate in English or French and have access to a telephone and the internet. HardHat RCT participants were interviewed at baseline, at 3 months, and at 6 months. Following the baseline interview, participants were randomized to the wait-list control group or the intervention group:

1. Wait-list control group: participants gained access to the HardHat program following their final (6 months) interview
2. Intervention group: participants were given immediate access to the HardHat program.

HardHat participants received incentives (about Can $25 [US $19] gift cards) for completing each of the baseline, 3-month, and 6-month interviews. Every other week, participants in the intervention group received email reminders to engage in the HardHat program. Unlike the BroHealth RCT participants, HardHat participants were also incentivized to complete program sessions. For each session completed, participants earned 100 Green Shield Canada points and were given a ballot for a monthly draw to win a US $100 gift card. The Green Shield Canada points could be used to buy additional ballots for the chance to win rewards such as gift cards.

### Sample Description

The original qualitative study design entailed a plan to interview a random sample of the BroHealth RCT intervention group completers to ask them about their experience using the BroHealth program and what aspects of the program they found effective. However, after the initial month of both the BroHealth and HardHat RCTs, we noted that the use of the web-based programs was low among participants in both BroHealth intervention groups and the HardHat intervention group. The sampling plan was shifted to encompass a random sample of participants from these 3 intervention groups; we asked these participants about their perceptions of the effectiveness of their respective web-based programs as well as about what might motivate them to use web-based programs.

A total of 77 participants provided data for this study. Sixty-one participants were from the BroHealth intervention and were organized into 2 groups as follows: BroHealth qualitative group 1 comprised 41 participants who were randomly selected from either of the BroHealth intervention groups (BroHealth intervention group 1=370 participants and BroHealth intervention group 2=374 participants) because they had logged in to BroHealth a maximum of one time during the RCT and were interviewed 2 to 10 months after commencing the program; BroHealth qualitative group 2 comprised 20 participants who were also randomly selected from either of the BroHealth intervention groups, regardless of whether and to what extent they had used the BroHealth program. The BroHealth qualitative group 2 participants were interviewed following the completion of the RCT. Rounding out our sample, a third qualitative group comprising 16 HardHat participants was randomly selected from the HardHat intervention group (HardHat intervention group=103 participants) because they had logged in to HardHat a maximum of one time. HardHat participants were interviewed 1 month into the RCT.

### Data Collection

Semistructured telephone interviews were conducted with participants, and interview duration ranged from 2 to 35 min and averaged approximately 9 min. Interviews were audio recorded and transcribed verbatim. All interviews included questions about aspects of the web-based programs that participants found effective, why they did not use the programs, and what did or would motivate program use. The interviews with the BroHealth group 2 participants tended to be lengthier, as these interviews included questions about experience with the RCT overall. The qualitative interview guides are included in the Multimedia Appendix 1.

### Sample Size Justification

We sought to capture a full range of both aspects of participants’ perceptions of the effectiveness of the web-based programs as well as factors that would motivate the use of such programs. As such, we were guided in our sample sizes by the principle of *data saturation*. Although aligning with Saunders et al [36] (among others) to recognize the multidimensional and contested nature of saturation, in our study, we aimed for data saturation, which we defined as “the degree to which new data repeat what was expressed in previous data.” In their discussion and review of guidelines for determining sample sizes needed to achieve *saturation*, Sim et al [37] presented sample size recommendations from an array of qualitative methodologists. Although saturation is contingent on the goals of the study being conducted, Sim et al [37] note that for studies that entail interview data collection, methodologists tend to argue that saturation can be achieved through interviews with as few as 10 and as many as 40 participants. Guided by these numbers, and in light of the particular but novel nature of our study focus, we began with a relatively large sample of 41 participants in our first group (BroHealth group 1). Well before interview 41, interviews became repetitive and did not provide new information. Consistent with our inductive analysis approach, for our second group (BroHealth group 2), we conducted 20 interviews; before the 20th interview, interviews became repetitive both within our second group and between our first and second groups. Accordingly, we reduced our sample size slightly for group 3 (HardHat group) to 16 participants, and we again found data saturation both within and between groups.

### Data Analysis

For this inductive content analysis, we began data analysis concurrent with interview data collection. After each interview, the recording was transcribed and reviewed by the research team. We then followed Elo and Kyngas’ [33] steps of *open coding* and *creating categories*. These steps began with immersion in the data achieved as a researcher read and reread interview transcripts. This researcher then open coded by rereading transcripts and highlighting pieces of data—words, phrases, or sentences—that reflected meanings in response to our interview questions about aspects of the web-based program that participants considered effective and factors that would motivate participants’ use of web-based programs. At this stage, this researcher created as many codes as necessary to reflect the meanings manifested in the interview content. Following this open coding, codes were grouped and collapsed into higher-order categories that captured the overall data set [38,39]. Transcript data and corresponding codes and categories were organized using NVivo 12 software.

### Trustworthiness

We enhanced the trustworthiness of our findings by using the triangulation of investigators in our analysis. Specifically, author 2 (MN) conducted a brief analysis by reading select transcripts and providing a summary of key issues evident in the transcripts. All transcripts were then reviewed and coded by author 1, looking for important pieces of data. Authors 1 (HE) and 2 (MN) reviewed both analyses for the similarity of issues identified in the transcripts. A third researcher who was external to this project was brought in to ensure that the broader categories were a fair representation of the codes that were identified. The 3 researchers then discussed and reached consensus about any inconsistencies in codes and categories.

This project was approved by the Research Ethics Board of the Royal Mental Health Centre in Ottawa, Canada.

## Results

### Sample Demographics

Of the 77 participants, 70 were males and 7 were females. Response rates in the form of the number of prospective participants contacted versus the number who agreed to participate in the interviews are depicted in Table 1.

The average age of the participants was 40.6 years (SD 1.34) and ranged from 20 to 65 years. The average risk of depression in the next 4 years was 23.1% and ranged from 7% to 87%. We generated 4 categories pertaining to the perceived effectiveness of the web-based mental health programs: awareness of health, functionality and medium, content, and coaches. We generated 5 categories pertaining to what motivates the use of web-based mental health programs: reminders and incentives, promotion of the programs, appropriate medium and functionality, perceived need, and questioning how or if motivating use is possible. Table 2 shows a summary of all categories generated through the content analysis.

**Table 1:** Response rates of each group of qualitative interviews.

Interview groups	Population (n)	Contacted, n (%)	Interviews, n (%)
BroHealth low usage	744	81 (10.9%)	41 (5.5%)
BroHealth after randomized controlled trial	744	101 (13.6%)	20 (2.7%)
HardHat	103	61 (59.2%)	16 (15.5%)

**Table 2:** Summary of the categories that were found through content analysis divided into their respective topics.

Topics	Categories
Effectiveness	Web-based programs increase awareness of mental health Perceived program functionality and the internet as a medium Contrasting perceptions of content effectiveness Coaches increase perceived effectiveness of programs
Motivators	Providing reminders and incentives increases motivation Promotion of web-based health programs increases motivation Providing appropriate medium and functionality increases motivation Perceived need for use increases motivation Motivating the use of web-based programs may be impossible

### Perceived Effectiveness of Web-Based Mental Health Programs

#### Web-Based Programs Increase Awareness of Mental Health

The most common benefit of web-based mental health programs identified by participants is that such programs improve mental health awareness. Some participants spoke positively about increased self-awareness resulting from the use of the programs. For instance, “...it forced me through some of the issues that I am having” and “I think it helped just be aware of some of the problems writing them down. You know, I just didn't really focus on them that much.”

Participants appreciated being aware of a resource that they could count on to support their needs currently and in the future. One participant explained:

I’d like to have it as a resource I can get to because there are times where like work, life and what not is more stressful than normal...and I want to make sure that I have help when I need it.

Participants believed that web-based programs are especially beneficial for individuals who are unwilling or financially unable to seek professional help.

Moreover, participants believe that web-based mental health programs can increase society’s awareness of mental health. Participants viewed the web-based programs as a “step in the right direction” in terms of reducing stigma and increasing access to help, especially for men:

...it also makes it a nice tool for people who don't want to talk about mental health among men. It's still a big stigma right now. Not a lot of people that want to admit to it, but it’s nice that it’s there.

#### Perceived Program Functionality and the Internet as a Medium

Functionality and medium were important considerations with respect to the perceived effectiveness of web-based health programs. Participants expressed conflicting views about using the internet. On the one hand, participants endorsed the idea of having 24/7 access and an increased level of privacy. For instance, one participant mentioned:

I would say that that makes it effective to me because it's available to me at 1 o' clock in the morning if I wake up and I can't sleep...or feeling anxious about something, that's an advantage to uh, to be able to look at something and at that time.

Another participant said:

I assume a lot of people who are in my situation are in a crazy schedule so like you know the ability to do something on your own time and not always have appointments booked for you is good.

On the other hand, participants mentioned having procrastinated because there was nobody to ensure their participation. Furthermore, many participants had limitations to their computer use for reasons such as long hours on the computer for work, not being comfortable with computer use, and not having internet access. For instance:

I work on computers all day in my job so to then after sit on a computer um you know, when I walk away from my work I cannot just spend a lot of time on computers.

Although there was little reference to specific aspects of web-based program functionality, most participants were happy with the overall functionality and ease of use of both programs. However, some participants in both programs expressed finding it difficult to navigate the websites:

It was okay, for the first little bit it might have been a little bit confusing and not really sure where to go, what I’m looking for.

Some participants also noted technical issues such as broken links that may inhibit the effectiveness of the program.

#### Contrasting Perceptions of Content Effectiveness

Participants had varying opinions regarding the effectiveness of the content of the web-based mental health programs. Some found the content to be appropriate, detailed and engaging, and effective at decreasing stress. One participant remarked:

What was helpful is that, I do need to get back to listing what my challenges and goals are.

Conversely, some participants did not find the program content useful for the following reasons: the content was too similar to other resources, the content was not relevant to the user, the program had a poor flow, and the program lacked appropriate information on mental health. One participant stated:

after a half a dozen times I found that the content was redundant with what I had already gotten from my healthcare provider.

Some participants believed that the amount and type of content were comprehensive and covered a wide area related to mental health, whereas other participants felt there was too much information and that *“*...there was lot of text so sometimes it became a bit heavy,” discouraging them from using the program.

Participants also recommended having personalized profiles to allow them to more quickly and easily narrow in on appropriate resources. Participants who believed the content to be appropriate and helpful cast their feedback in broad terms such as *“*It was engaging. It was appropriate*.*” The few people who gave specific positive feedback appreciated the elements that they could adopt and implement in their daily lives, thus increasing their personal benefits of program use. Participants also suggested that periodically updating the content would have encouraged ongoing use.

#### Coaches Increase the Perceived Effectiveness of Programs

Although 45 participants had access to a coach (intervention group 2 of BroHealth and HardHat intervention group participants), very few used their coach. Of the 29 participants in BroHealth intervention group 2 who had access to a coach, only 2 utilized the coach and they believed that it was a great feature of the program:

I think I called at least 6 to 8 times and each time I was really happy to talk to him...to bring me back and to give me good advice and to take me back to planet earth a little.

None of the 16 HardHat intervention group participants used their coach. Most were not aware that there was a coach included in the program or were unsure of the role of the coach. A few participants expressed not feeling comfortable talking with a coach because they were unsure about what to say. Participants who were either not given access to a coach (intervention group 1 in the BroHealth RCT) or who did not use their coach speculated that having a counselor to talk to would be an added benefit of the program. One participant compared the availability of BroHealth coaches with the availability of counselors in another program:

...they utilize an interesting way of connecting live counsellors with people who are actively looking to chat right now. And that's the only thing that wasn't there.

HardHat participants also believed that coaches would benefit them:

I think that getting that personal connection with the coach to be able to have that personal connection and possibly be able to relate to them.

Participants from both programs recommended having counselors that they could speak to via text, phone, or in person by appointment or on a 24/7 basis. Participants believed this would improve the web-based programs in 2 ways: (1) by keeping participants accountable by scheduling appointments and (2) by giving them someone to talk to if they need extra help with their mental health.

### What Motivates the Use of Web-Based Mental Health Programs

#### Providing Reminders and Incentives Increases Motivation

Most participants believed that the reminders were beneficial in increasing their program use:

…when I got an e-mail from you before that sort of was a trigger to take a look.

At the same time, many participants did not recall receiving these reminders, thus limiting the perceived effectiveness of reminders. Many participants believed that text message reminders, rather than email, would be a more effective way to motivate program use:

I don't always check my emails, it's not something that I necessarily sit down and do on a daily basis. Everybody always has their phone with them.

Participants also acknowledged that the follow-up interviews conducted as part of the RCTs served as reminders to use the programs:

A lot of it was after interviews that I've done previously. It was a good reminder that the website was there.

Participants in the HardHat RCT received incentives to engage in the program, whereas participants in the BroHealth RCT did not. Participants who received incentives and participants who did not receive incentives indicated that incentives were a good motivator for program use. For instance, one participant commented:

Something like points or rewards for progress as you work through it kind of thing might be a motivator to go back to it.

#### Promotion of Web-Based Health Programs Increases Motivation

Overall promotion and marketing of web-based mental health programs are major motivators to use the program. Participants believed that better promotion, and thus awareness and knowledge of the program, is the first step in motivating program use. For instance, one participant said:

It really is just trying to motivate the person to click it once, I suppose, and go on there.

Moreover, participants believed that having a good understanding of the program and its benefits before starting the program would motivate use. Various ways to promote use to people not already using the web-based programs were described by participants, including emails, text messages, word of mouth, and social media. One participant suggested using employers and workplaces to distribute information about the program, ensuring that a support system in the workplace is in place, and even having the program integrated within the workplace. Another participant said:

...it would be nice if things were within the workplace. In the workplace there's more structure. A lot of my stress derived from being self-employed and not having uh the workplace environment there to support me.

#### Providing the Appropriate Medium and Functionality Increases Motivation

The type of medium and functionality of the web-based programs were significant motivators. Many participants mentioned the importance of personal preference, noting that younger generations tend to be drawn to web-based media. Participants conveyed the importance of the medium fitting into their everyday life to motivate them to use the program. For instance, having notifications on their social media accounts and/or being able to access the program as an app on their mobile device were motivators. One participant explained:

whatever kind of sites that a person connects through...so you know if I get, if I get Facebook updates like that.

Many participants mentioned that being able to talk to a person (via text messaging, phone, or in person) in some capacity is beneficial for web-based mental health programs and would motivate use by increasing accountability. Ease of use (ie, “quick, easy, and convenient”) was also a motivator to use the programs, as these characteristics allow the programs to fit into the busy lives of the participants.

#### Perceived Need for Use Increases Motivation

Participants perceiving that they need the program and perceiving the program as beneficial are major motivations for program use. Many nonusers anticipated that they would use web-based mental health programs in the future if they perceived a need for it. These nonusers did not believe that they were currently going through a hard time or having any symptoms; thus, they did not believe they had a personal need for the program.

Participants who did perceive themselves as having a need for the program were more motivated to continue using the program when program content was relevant to their lives. For instance, one participant said:

That one time I did go into it, I liked what I saw so I'm going to be going back.

Having content relevant to participants’ experiences makes participants more likely to achieve the benefits of the program, in turn, improving motivation to use the program on a continuous basis. Updating the content may also lead to continued use.

#### Motivating the Use of Web-Based Programs May Be Impossible

Although motivating the use of web-based mental health programs is an important endeavor to ensure the use of mental health programs, many participants were simply unsure of how to do so or felt that motivating use was not possible. For instance, one participant stated:

...that's a really good question and if it was an easy answer, I wish it was an easy answer.

Others felt that the barriers to using such programs, such as lack of prioritization and stigma, are unsurmountable. For example, one participant explained:

when it comes to stacking priorities, how would you get around that, I really don't know.

Another participant commented:

...in my opinion, probably can't...I just come from a different school of thought I guess. That's just not a man thing to do.

## Discussion

### Principal Findings

Web-based mental health programs are valuable in preventing and treating depression [20-22]. To increase the use of prevention programs, it is important to understand what motivates the use and the perceived effectiveness of various components of the programs. Functionality and medium, awareness of mental health, and appropriate content were identified in this study as important factors for judging the perceived effectiveness of web-based mental health programs. Motivators to use such programs include providing reminders and incentives; promoting the programs (including endorsement by employers); providing appropriate medium, functionality, and content; and having a perceived need. At the same time, some participants were unsure of how they could be motivated to use the programs or felt that there may be no way to do so.

Past literature describes many benefits of using web-based mental health programs, including convenience, accessibility, and cost-effectiveness [24]. Furthermore, having both increased awareness of one’s mental health and good mental health literacy (ie, having knowledge about symptoms, treatments, and resources) is an important element of depression prevention [40,41]. Increased awareness reduces stigma and encourages individuals to access mental health services before the onset of depression. Our study echoes these findings, as mental health awareness was perceived as the largest benefit of the programs that we investigated.

### Implications for Future Programs

Despite the benefits described earlier, adherence to web-based programs for mental health is low. Thus, the question is, how can we motivate people to use web-based health programs for mental health? Furthermore, how can we motivate people to use web-based programs before developing depression?

#### Promoting Web-Based Mental Health Programs

The first and, arguably, the hardest step is to convince people to use such programs for the first time. To convince people to use these programs, people need to (1) have the knowledge that such programs exist and (2) believe that they have a need for the services provided by the web-based mental health programs. Useful marketing of the programs was a very strong motivator uncovered in this study. Primary care providers are the number one source of mental health information [42], but many people are reluctant to see their physician about mental health problems, especially for prevention. As a result, a large proportion of people are not receiving the resources they need. The results of this study indicate that men who are reluctant to see mental health professionals and use mental health resources may be more open to discussing resources with people close to them. Although men are often afraid of stigmatization, recent studies have reported that many men have a small social circle with whom they feel they can share their concerns [43]. Men are also very willing to support the mental health of other people in their lives [43]. This close social circle/familial support adds an avenue through which to promote the initiation of web-based mental health programs.

Mental health programs within the workplace have been reported in the literature as decreasing stigmatization of poor mental health [44]; workplace mental health programs may contribute to an increase in web-based program use. This study uncovered a complementary finding that participants may be more likely to use web-based mental health programs if these programs are somehow incorporated into the workplace setting. In addition, having a program in the workplace was found in this study to increase feelings of mental health support. Thus, promoting web-based programs in the workplace may help to increase awareness about the program and more effectively encourage initial and ongoing participation.

Once a person has decided to initiate the use of a mental health program, they need to complete it to reap the full benefits [45]. Many methods have been noted in the literature to increase adherence to web-based programs.

#### Improving the Continued Use of Web-Based Mental Health Programs

Our study found that appropriate content was an important factor for increasing the perceived effectiveness and, consequently, the increased use of web-based mental health programs. However, as content needs often vary significantly from person to person, the participants in this study postulated that having individually tailored content may motivate the continued use of a web-based program. Moreover, meta-analyses and systematic reviews have shown that having tailored content can decrease symptoms of depression in study populations [46,47]. Having future programs tailored to a specific group or having the ability to tailor to each specific user would be beneficial to improve adherence and effectiveness of the program. In addition, the results of this study suggest that tailoring the web-based mental health program to people with lower mental health literacy would make the content less redundant. Lustria et al [46] found that computer assessments could be used to narrow down the content to that users’ specific needs. Future research should focus on how to effectively tailor content to specific groups.

In addition, reminders not only increase adherence to [48] and perceived effectiveness of [49] web-based programs but also help foster a positive attitude toward them [50]. In this study, the reminder emails motivated program use. However, email reminders were often discarded immediately by participants without being read. Many participants speculated that reminders sent via text messages may be a better format. Having a direct link to the program to make it faster and easier for participants was endorsed by this study. Although studies have found results consistent with this finding [51], more research is needed to find the optimal medium for reminders to motivate program use.

The use of the internet as a medium was a polarizing topic, as web-based programs are not preferred by everyone. Although some participants believe using the internet as a medium is ideal because of privacy and convenience, others preferred alternate media as they had limited computer use. This discrepancy is consistent with other studies [52]. For instance, a study by Smail-Crevier et al [53] found that younger generations are more likely to engage with web-based programs compared with older generations. Another study found that high computer/internet use is negatively associated with the use of web-based mental health programs [54]. Moreover, having a live person to speak to is associated with increased use and completion of the program [54]. Agreeing with the literature, the results from this study demonstrate that having access to someone to speak to may make the programs more effective and meaningful to users and thus could increase motivation and adherence to future web-based programs. The results of this study indicate that although coaches or guides in programs could potentially be effective, unless participants are aware that they have access to a coach and understand the role of the coach, they are unlikely to use it.

#### Impact of Society on Web-Based Mental Health Programs

Although the methods to motivate use described above have been found to be effective, there are many individuals who are unsure of how to motivate their own use or who simply do not believe that it is possible to motivate people in general because depression and help-seeking remain highly stigmatized [55-58]. Specifically, at-risk individuals tend to have lower mental health literacy and are less likely to seek treatment [59]. The Health Belief Model suggests that a person will only take action to prevent disease if (1) they believe they are vulnerable to the disease, (2) the disease is severe enough to negatively affect them, and (3) there is a benefit to the prevention that outweighs the cost [60]. These findings have been echoed in previous research on depression [61-63] and in our study, as participants were more likely to use the programs if they believe that they needed it.

### Limitations

This study is limited in several ways. First, given the specific and detailed nature of this study, we make no claims that our findings can be extrapolated to larger populations or to other web-based mental health programs. Second, there is a chance that a different research team with different backgrounds would generate different categories from our data. To offset this limitation, multiple researchers collaborated and achieved a consensus on the analysis. We note further that some participants were interviewed a few months after they had used the program and thus may have had difficulty with recall. Our study did not examine how demographics, such as age, income level, and occupation, could influence participants’ perceptions about web-based mental health programs. Finally, very few participants engaged with their coaches. As a result, the views that they expressed about coaches were often not based on personal experience and should therefore be interpreted with caution.

### Conclusions

In conclusion, awareness of one’s own mental health is a benefit of using web-based mental health programs. The content and functionality of the programs are important factors when assessing perceived effectiveness. However, perceived effectiveness varies from person to person, and what may be effective for one person may not be effective for another. Access via chat to a real person and being able to customize content to fit the users’ personal needs are improvements that could increase the perceived effectiveness and use of similar programs. Motivating people to use such programs is difficult; even current program users are unsure of the best ways to motivate use. Motivators include providing reminders or incentives, promoting the programs, providing appropriate medium and functionality, and perceived need, which, if adequately incorporated into web-based program design and implementation, may lead to increased use.

## Appendix

Multimedia Appendix 1 Qualitative interview guide.

## Abbreviations

CBT: cognitive behavioral theory
MDE: major depressive episode
RCT: randomized controlled trial
